# Is pentobarbital safe and efficacious in the treatment of super-refractory status epilepticus: a cohort study

**DOI:** 10.1186/cc13883

**Published:** 2014-05-21

**Authors:** Deborah Pugin, Brandon Foreman, Gian Marco De Marchis, Andres Fernandez, J Michael Schmidt, Barry M Czeisler, Stephan A Mayer, Sachin Agarwal, Christine Lesch, Hector Lantigua, Jan Claassen

**Affiliations:** 1 Division of Critical Care Neurology, Department of Neurology, Columbia University, College of Physicians and Surgeons, 177 Fort Washington Avenue MHB 8 Center, Room 300, New York, NY 10032, USA; 2 Department of Pharmacy, New York-Presbyterian Hospital, New York, NY, USA; 3 Comprehensive Epilepsy Center, Department of Neurology, Columbia University, College of Physicians and Surgeons, 177 Fort Washington Avenue MHB 8 Center, Room 300, New York, NY 10032, USA

## Abstract

**Introduction:**

Seizures refractory to third-line therapy are also labeled super-refractory status epilepticus (SRSE). These seizures are extremely difficult to control and associated with poor outcome. We aimed to characterize efficacy and side-effects of continuous infusions of pentobarbital (cIV-PTB) treating SRSE.

**Methods:**

We retrospectively reviewed continuous electroencephalography (cEEG) reports for all adults with RSE treated with cIV-PTB between May 1997 and April 2010 at our institution. Patients with post-anoxic SE and those receiving cIV-PTB for reasons other than RSE were excluded. We collected baseline information, cEEG findings, side-effects and functional outcome at discharge and one year.

**Results:**

Thirty one SRSE patients treated with cIV-PTB for RSE were identified. Mean age was 48 years old (interquartile range (IQR) 28,63), 26% (N = 8) had a history of epilepsy. Median SE duration was 6.5 days (IQR 4,11) and the mean duration of cIV-PTB was 6 days (IQR 3,14). 74% (N = 23) presented with convulsive SE. Underlying etiology was acute symptomatic seizures in 52% (N = 16; 12/16 with encephalitis), remote 30% (N = 10), and unknown 16% (N = 5). cIV-PTB controlled seizures in 90% (N = 28) of patients but seizures recurred in 48% (N = 15) while weaning cIV-PTB, despite the fact that suppression-burst was attained in 90% (N = 28) of patients and persisted >72 hours in 56% (N = 17). Weaning was successful after adding phenobarbital in 80% (12/15 of the patients with withdrawal seizures). Complications during or after cIV-PTB included pneumonia (32%, N = 10), hypotension requiring pressors (29%, N = 9), urinary tract infection (13%, N = 4), and one patient each with propylene glycol toxicity and cardiac arrest. One-third (35%, N = 11) had no identified new complication after starting cIV-PTB. At one year after discharge, 74% (N = 23) were dead or in a state of unresponsive wakefulness, 16% (N = 5) severely disabled, and 10% (N = 3) had no or minimal disability. Death or unresponsive wakefulness was associated with catastrophic etiology (p = 0.03), but none of the other collected variables.

**Conclusions:**

cIV-PTB effectively aborts SRSE and complications are infrequent; outcome in this highly refractory cohort of patients with devastating underlying etiologies remains poor. Phenobarbital may be particularly helpful when weaning cIV-PTB.

## Introduction

Seizures do not respond to first- and second-line therapy in 9 to 40% [1-4] of patients with status epilepticus (SE). This condition, known as refractory status epilepticus (RSE), is associated with high morbidity [3-6] and is typically treated with anesthetic agents such as midazolam or propofol [7]. Among these patients, 10 to 15% [4] fail to respond to third-line therapy, and are considered to have super-refractory SE (SRSE) [8]. These patients are not well-studied, and in the absence of randomized clinical trials treatment is controversial [9]. Continuous intravenous (IV) infusions of pentobarbital (cIV-PTB), a mainstay of RSE treatment for the past 50 years, have been used less frequently as third-line therapy in favor of midazolam or propofol. However, many protocols recommend cIV-PTB for treatment of RSE refractory to midazolam or propofol infusions [7,10]. We sought to describe the use of cIV-PTB in patients with SRSE. More specifically, we aimed to assess the efficacy of cIV-PTB in terminating seizures, the occurrence of withdrawal seizures, and the safety of using cIV-PTB in the modern era of ICU care.

## Methods

In this retrospective cohort study, patients with SE admitted to Columbia University between May 1997 and April 2010 were identified from continuous electroencephalography (cEEG) reports. All adults with SRSE monitored with cEEG and treated with cIV-PTB were included in the study. Patients with post-anoxic SE or those receiving cIV-PTB for reasons other than RSE were excluded. Medical records were reviewed for clinical details. The Institutional Review Board of Columbia-Presbyterian Medical Center [11] approved this study and waived the need for informed consent as it was a retrospective chart review study.

### Definitions

RSE was defined as ongoing or recurrent seizures without recovery of consciousness or return to baseline for at least 30 minutes, and no response to first- and second-line anti-epileptic drugs (AED) [7]. SRSE was defined as ongoing or recurrent SE 24 h or more after starting continuous infusion midazolam or propofol, and included cases in which SE recurred upon reduction or withdrawal of anesthesia [8]. PTB failure was defined as a change to another AED due to failure of seizure control despite anesthetic doses of cIV-PTB (doses of at least 1 mg/kg/h) or intolerability to PTB side effects. Withdrawal seizures were defined as the recurrence of seizures during or within 48 h after tapering or withdrawal of cIV-PTB [11,12]. SE etiology was classified as acute symptomatic, remote symptomatic, progressive symptomatic or idiopathic/cryptogenic. We also identified patients with so-called catastrophic underlying etiology if the life expectancy due to the underlying etiology was likely less than 90 days. Seizure semiology was classified as generalized convulsive or non convulsive, assessed according to the earliest description prior to treatment administration. The onset of SE was considered to be the beginning of clinical seizure activity or a significant or sudden decline in neurologic function. Termination of SE was defined as cessation of seizure activity on cEEG, regardless of recovery of consciousness, without seizure recurrence for at least 48 h after cessation of cIV-PTB. The levels of AEDs were checked for all patients and confirmed to be in a therapeutic range before starting cIV-PTB.

### Patient management

EEG monitoring was performed for all patients at least 24 h prior to initiation of cIV-PTB and continued through the weaning period except for patients who died before completion of treatment. Patients with SE were treated according to previously published institutional protocols [13-15]. SE treatment was evaluated according to current international guidelines and considered adequate if it included intravenous administration of at least one benzodiazepine, plus phenytoin (PHT), levetiracetam (LEV) or valproate (VPA), and was followed by continuous IV infusion of midazolam (cIV-MDZ) or propofol (cIV-PRO). Pentobarbital was administered as a 10 to 15 mg/kg loading dose followed by continuous infusion of 0.5 to 4.0 mg/kg/h and the dose was adjusted to achieve seizure control often resulting in a suppression-burst pattern (SBP) on cEEG.

### Data acquisition

Demographic data, medical history including prior epilepsy, admission neurologic findings, SE etiology and semiology, medication history prior to admission and medications given in the hospital (specifically AEDs, including doses and levels), cEEG findings, and hospital complications (including potential side-effects from SE therapy: hypotension requiring pressors administration or modification, arrhythmia, myocardial infarct, pulmonary embolism, acidosis, fluid overload, and multiorgan failure) were recorded. Data were collected through analysis of discharge summaries, clinical notes, and laboratory results. We recorded functional outcome at discharge and at 1 year, which was considered poor if the patient died, was in a state of unresponsive wakefulness, or had severe disability (Glasgow outcome scale (GOS) 1 to 3) and good if the patient had no or minimal disability (GOS 4 to 5). As part of a larger study, data were also collected on patients with RSE. These were used as a comparison group.

### Statistics

Data were analyzed using commercially available software (STATA version 11, College Station, TX, USA). Categorical variables were compared using the Chi-Square test or the Fisher exact test, as appropriate. Continuous variables were compared using the two-tailed *t*-test or Mann-Whitney *U*-test for non-normally distributed data. After identifying variables associated with outcome in the univariate analysis we performed a binary logistic regression analysis to identify independent outcome predictors.

## Results

### Demographics

During the study period, 147 patients presented with RSE, 21% (31/147) of whom fulfilled criteria for SRSE treated with cIV-PTB during cEEG. In the SRSE group, mean age was 48+/-20 years and 56% (17/31) were female (see Table 1). Of the patients, 26% (8/31) had a history of epilepsy, including two patients with Lennox-Gastaut and one patient with cortical dysplasia. Initial seizure semiology was convulsive in almost two thirds and non convulsive in one third of SRSE patients. Of the patients with acute etiology, 75% (12/31) had encephalitis: 26% (8/31) had catastrophic etiologies including 13% (4/31) with complicated astrocytoma (World Health Organization grade III), 6% (2/31) with end-stage pancreatic cancer, and one each with acute rejection of cardiac transplant and meningeal carcinomatosis. In comparison, patients with RSE who did not progress to SRSE were older (odds ratio (OR) 0.96;CI 0.94-0.98), and more frequently had intracerebral hemorrhage (OR 0.09, 95% CI 0.01, 0.69). Patients with SRSE were more likely to have encephalitis as the underlying etiology of SE (OR 4.35, 95% CI 1.71, 11.09).

**Table 1:** Demographic and etiology

	**SRSE n = 31**	**RSE n = 116**	**Odds ratio (95% CI)**	***P*-value**
Age, years^1^	48 (+/-20)	61 (+/-17)	0.96 (0.94, 0.98)	0.001^*^
Women, n (%)	17 (55)	78 (67)		
Race, n (%)				
White	15 (48)	52 (45)	-	-
Non white	16 (52)	64 (55)	-	-
History of epilepsy, n (%)	8 (26)	38 (33)		
Etiology, n (%)				
Acute	16 (52)	70 (60)	-	-
Encephalitis	12 (35)	13 (11)	4.35 (1.7, 11.09)	0.002^*^
Intracerebral hemorrhage	1 (3)	31 (27)	0.09 (0.011, 0.69)	0.021
Stroke	1 (3)	4 (3)		
Toxic-metabolic	1 (3)	11 (9)		
Traumatic brain injury	1 (3)	11 (9)		
Progressive	10 (30)	35 (30)	-	-
Neoplasia	4 (10)	7 (6)		
Degenerative	1 (3)	6 (5)		
Epilepsy	5 (16)	22 (19)		
Idiopathic/cryptogenic	5 (16)	11 (9)		
Catastrophic etiology, n (%)	8 (26)	NA	-	-
Length of SE, days^2^	6.5 (4, 11)	NA	-	-
Type of SE, n (%)				
Convulsive SE	23 (74)	66 (57)		
NCSE	8 (26)	50 (43)		

Data are given as number (n) (%), ^1^mean (+/- SD), or ^2^median (IQR). Catastrophic etiology is defined as life expectancy due to underlying etiology less than 90 days. The odds ratio for encephalitis associated with SRSE was 2.91 (1.08, 7.85) after correcting for age. ^*^Age and encephalitis were independently associated with SRSE. NCSE, non-convulsive status epilepticus; SE, status epilepticus; RSE, refractory status epilepticus; SRSE, super-refractory status epilepticus; NA, not applicable.

### AED treatment

All patients admitted with SRSE who received cIV-PTB received first-, second-, and third-line therapy. Most patients received a combination of a BZD and PHT (25/31), followed by combinations of either BZD plus VPA (4/31) or BZD plus LEV (2/31). Administered AED medications included PHT/fosPHT in 80% (25/31), VPA in 67% (21/31), LEV in 61% (19/31), phenobarbital in 35% (11/31), topiramate in 26% (8/31), lacosamide in 9% (3/31), and ketamine in 9% (3/31) of patients. Midazolam was the primary third-line choice in 94% of patients (29/31); 13 of these later received propofol in addition to midazolam, and the remainder received only propofol infusions (6%; 2/31). At the time of starting cIV-PTB, 93.5% (29/31) of the patients had received at least four AEDs, up to a maximum of eight AEDs.

### cIV-PTB treatment

cIV-PTB was instituted within 6 days (IQR 2, 10) of the onset of RSE. A maintenance dose of 0.5 to 1.7 mg/kg/h of cIV-PTB was sufficient to maintain SBP in 64% of patients, but increases of up to 2.0 to 3.7 mg/kg/h were required for 36% of patients. The total duration of cIV-PTB was 6 days (IQR 3, 14). PTB successfully stopped SE in 90% (28/31) of the patients (Table 2). Two patients with catastrophic etiologies and one with encephalitis were considered PTB failures. Withdrawal seizures were observed in 48% (15/31) of patients while attempting to wean cIV-PTB. PTB was restarted in seven patients, all of whom responded to restarting the medication. Phenobarbital was added in 12 of the 15 patients with withdrawal seizures, which allowed successful weaning of cIV-PTB. On cEEG, 90% (28/31) of patients achieved SBP, which lasted more than 72 h in 56% of patients (17/31): 9% of patients (3/31) developed complete EEG suppression on initiation of cIV-PTB and dosing was reduced with restoration of SBP. The overall length of the SBP correlated with control of SRSE by PTB (*P* = 0.01). After termination of SE, the SBP evolved most frequently to generalized periodic discharges (GPDs) (46% of patients, n = 13) or lateralized periodic discharges (LPDs) (16% of patients, n = 5). Patients without GPDs or LPDs had severe attenuation (n = 5), persistent SBP (n = 1) or markedly slow background (n = 2). Periodic discharges were associated with a longer duration of PTB drip and a longer SBP duration, but were not predictive of seizure relapse after stopping cIV-PTB.

**Table 2:** Anti-epileptic drug data

**Variable**	**Number (%) or median (IQR)**
Total number of patients	31 (100%)
Number of AEDs before pentobarbital	
3	2 (6)
4	8 (26)
5	7 (23)
6	8 (26)
7	4 (13)
8	2 (6)
Response to pentobarbital, patients	28 (90)
Length of pentobarbital drip, days	6 (3, 14)
Length of status epilepticus before pentobarbital initiation, days	6 (2, 10)
Withdrawal seizure after interruption of pentobarbital, patients	15 (48)
Withdrawal seizure requiring pentobarbital restart, patients	7 (47)^1^
Phenobarbital to wean pentobarbital, patients	12 (80)^1^

^1^Percentages refer to number of patients with withdrawal seizure after interruption of pentobarbital (n = 15). Response to pentobarbital is defined as status epilepticus that was completely controlled by the therapy. Withdrawal seizures are defined as recurrence of status epilepticus during or within 48 h after the tapering or withdrawal of the therapy. AEDs, anti-epilectic drugs.

### Safety

**Figure 1:**
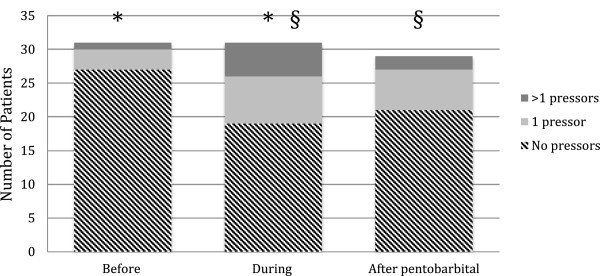
Vaspressors and pentobarbital. ^*^Comparison before/during pentobarbital (*P* = 0.016). ^§^Comparison during/after pentobarbital (*P* = 0.001).

Of the 31 patients, 11 had no side effects related to starting cIV-PTB (Table 3). Overall, ventilator-associated pneumonia (32%) and hypotension requiring pressors (32%) were the most frequently encountered complications during cIV-PTB treatment, followed by urinary tract infection (13%), deep venous thrombosis (10%) and ileus (10%). After initiation of cIV-PTB, the percentage of patients requiring vasopressors increased from 13 to 32% (Figure 1). In all cases hypotension responded to fluid administration and vasopressors, and did not require a switch in cIV-AEDs: 15% of the patients (n = 7) had other side effects such as neuropathy (n = 1), brain edema and bleeding following brain biopsy (n = 1), and sepsis which started before cIV-PTB (n = 3). One patient died of post-anoxic encephalopathy after a bradycardic arrest the day following cIV-PTB initiation, which occurred in the setting of several episodes of bradycardia requiring atropine administration prior to starting cIV-PTB. Another patient with N-methyl-D-aspartate receptor encephalitis developed propylene-glycol intoxication with severe acidosis, which resolved after cessation of cIV-PTB. All patients in the study were mechanically ventilated prior to starting cIV-PTB.

**Table 3:** Side effects that developed in 31 patients during treatment with pentobarbital

**Side effects**	**Number of patients (%)**
None	11 (35)
Ventilator-associated pneumonia	10 (32)
Hypotension	10 (32)
Urinary tract infection	4 (13)
Deep venous thrombosis	3 (10)
Ileus	3 (10)
Other	7 (23)

Patients may have had more than one side effect.

### Outcome

The duration of SE was 6.5 days (IQR 4, 11): 74% (23/31) of patients were referred from other hospitals, and 26% (8/31) were primarily admitted at our institution. The median ICU and hospital length of stay were 30 days (IQR 16, 48; minimum 6, maximum 141 days) and 48 days (IQR 32, 94, minimum 11, maximum 310 days), respectively. Catastrophic etiology as the underlying cause of SE was associated with death or unresponsive wakefulness at 1 year (*P* = 0.03). Age, duration of SE, International League Against Epilepsy (ILAE) etiology or SE type, and history of epilepsy were not associated with outcome in our cohort of SRSE treated with cIV-PTB. Of patients with catastrophic etiology, 75% (n = 5) were seizure-free until the end of monitoring with cIV-PTB (Table 4). However, two patients with catastrophic etiology did not respond to cIV-PTB and were considered PTB failures, both of whom died in the Neurological ICU. The length of SBP did not correlate with outcome at discharge or at one year. At discharge, 65% (n = 20) of patients were dead or in a state of unresponsive wakefulness (GOS 1 to 2), 32% (n = 10) had severe disability, and 3% (n = 1) had no or minimal disability (GOS 4 to 5). The mortality at discharge was 42% (n = 13). At one year, 62% (n = 18) had died, 15% (n = 5) were in a state of unresponsive wakefulness and 10% (n = 3) had a GOS of 4 to 5.

**Table 4:** Outcome in 31 patients

**Outcome at discharge**	**Number of patients (%)**
Outcome at discharge	
Mortality (GOS 1)	13 (42)
GOS 1 to 2	20 (65)
GOS 3	10 (32)
GOS 4 to 5	1 (3)
Outcome at 1 year	
Mortality (GOS 1)	18 (58)
GOS 1 to 2	23 (74)
GOS 3	5 (16)
GOS 4 to 5	3 (10)

GOS, Glasgow outcome scale.

## Discussion

This study confirmed the high morbidity and mortality of patients with SRSE treated with cIV-PTB; 42% were dead at discharge and only 10% had favorable recovery one year after discharge. However, favorable outcomes were possible, and treatment-associated morbidity was manageable in the ICU setting. Our cohort is unique as it focused only on SRSE patients, whereas previously published cohorts have been mostly a mix of patients RSE and SRSE [1,4,12,16,17].

### Efficacy

Of the studied SRSE patients, 90% were successfully treated with cIV-PTB. In previous studies, the efficacy of cIV-PTB in RSE ranged from 65% to 100%, but apart from a single series reporting 40 patients, the others included fewer than 18 patients [18-25]. Our results are consistent with a meta-analysis of patients with RSE treated with PTB in which 92% were seizure-free with PTB, compared to 80% with midazolam or 73% with propofol [11]. In our cohort, 90% of patients achieved SBP on cEEG, whereas the prevalence of SBP after treatment with cIV-PTB in prior studies has ranged from 38 to 60%. Most patients treated with cIV-PTB in those studies had been treated towards a goal of background suppression rather than SBP [6,13,18,20-26]. As the efficacy of cIV-PTB treatment in our cohort was comparable, we emphasize that SBP may be a reasonable goal for PTB therapy, and in particular, SBP lasting longer than 72 h may provide superior seizure control.

While weaning from cIV-PTB, 48% of patients developed withdrawal seizures which is comparable to previous descriptions (9 to 43%) [11,18-25,27]. At the same time, restarting PTB interrupted seizures in all of our patients, compared with 22% reported previously [8]. The addition of PTB allowed successful weaning of cIV-PTB in 80% of patients in our series. The high frequency of withdrawal seizures, affecting almost half of the patients, stresses the necessity for Ceeg, not only while starting cIV-PTB, but also while weaning it in order to be able to detect and treat these seizures as soon as possible.

### Safety

In contrast to other reports, we found fewer side effects in our cohort: 23% of patients did not have any side effects attributable to treatment with cIV-PTB. Compared with previously published data mostly on RSE, we found ventilator-associated pneumonia in 32% (versus 65 to 69%) [3,5], hypotension requiring fluid and vasopressors in 32% (versus 65 to 77%) [3,11], and urinary tract infection in 13% (versus 42 to 46%) [3,5]. One patient developed transient propylene glycol intoxication associated with cIV-PTB infusion. However, none of these complications influenced the outcomes, which is consistent with previous descriptions [11] In previous RSE studies including patients specifically treated with PTB, hypotension was reported in 65 to 100%, but most of these studies aimed to treat patients with a goal of EEG background suppression [18,20,22-25]. The only adverse event in this cohort was an isolated case of refractory bradycardia, although the patient had several episodes of bradycardia requiring atropine in the days before PTB initiation. In a controlled setting such as the ICU, the use of cIV-PTB to treat SRSE is safer than previously described, and the concern for its side effects should not limit its use except perhaps in circumstances of acute hemodynamic instability.

### Outcome

Mortality in our cohort was higher than previously described for SE (42% versus 7 to 39%) [1,3-5] with a wide range attributable to differences in study design (retrospective versus prospective) and inclusion criteria. Mortality rates of 42% reported in a prior cohort of long-lasting RSE, and 48% in RSE patients treated with cIV-PTB [1,3-5,11] are concordant with recent data that RSE, and particularly SRSE, carries a worse outcome than SE [4,16] This high mortality rate may reflect the selection of patients with SRSE who constitute a relatively ill cohort with higher prevalence of more malignant underlying etiologies and highly refractory seizures [28]. In addition, all our patients were mechanically ventilated, which in itself has been associated with higher mortality [29]. Only 3% of patients had little or no disability at discharge, improving to 10% at one year, confirming that SRSE is a severe disease with poor functional outcome compared to SE. Nevertheless, a small number of patients did well and improved with time, even with SE lasting for several days. None of the variables usually described to be related with outcome, such as age, duration of SE, or a history of epilepsy, correlated with outcome in our cohort [11,30-32]. The only variable predicting outcome in our series was a catastrophic underlying etiology. Three-quarters of patients with catastrophic etiology had control of SE with cIV-PTB, however; it is most likely that the underlying disease was the principle contributor to outcome in these cases. Our series was likely underpowered to detect differences in mortality based on age, duration of SE, or a prior history of epilepsy, but perhaps SRSE constitutes a subgroup of patients in whom these established predictors of poor outcome in RSE are less meaningful [5].

### Limitations

Our cohort is one of the largest sets of patients with SRSE treated with cIV-PTB, yet our analysis was still underpowered to detect differences in outcome; as such, we were unable to build a multivariate outcome model. This was a retrospective single-center study that excluded patients who received cIV-PTB prior to transfer where it may have been given as third-line treatment and our institutional protocol did not allow the use of cIV-PTB as third-line therapy. This limits the generalizability of our findings to RSE patients treated with cIV-PTB as third-line treatment, as reported in much of the older literature [18-25]. All RSE and SRSE patients at our institution undergo cEEG monitoring, which therefore does not represent a selection bias. The use of outside hospital records and the accuracy of patient records kept over such a prolonged time span may introduce information bias.

## Conclusions

Our study confirms that SRSE carries a poor prognosis but underlying catastrophic etiology was the only variable predictive of poor outcome in our cohort. CIV-PTB appears relatively safe to use in the ICU setting except in circumstances of acute hemodynamic instability. cIV-PTB is very effective to treat SRSE: 90% of our cohort were seizure-free after treatment with cIV-PTB. Withdrawal seizures were frequent (48% of patients) however, and cEEG is required to detect and treat them promptly, and PTB may be helpful in reducing the chance of withdrawal seizures. Ten percent of patients with SRSE may recover despite long duration of SE, therefore, the length of SE by itself should not inform discussions of goals of care except in cases with catastrophic etiology.

## Key messages

• Pentobarbital can be relatively safely used in the ICU environment.

• Pentobarbital is effective to treat super-refractory status epilepticus.

• Catastrophic etiology was the only variable associated with poor outcome. None of the other variables usually related to outcome in refractory status epilepticus were associated in our cohort with SRSE.

## Abbreviations

AED: Anti-epileptic drug; cEEG: continuous electroencephalography; cIV-MDZ: continuous infusion of midazolam; cIV-PRO: continuous infusion of propofol; cIV-PTB: continuous infusion of pentobarbital; GOS: Glasgow outcome scale; GPD: generalized periodic discharge; LEV: levetiracetam; LPD: lateralized periodic discharge; OR: odds ratio; PHT: phenytoin; RSE: refractory status epilepticus; SBP: suppression-burst pattern; SE: status epilepticus; SRSE: super-refractory status epilepticus; VPA: valproate. 

## Competing interests

SAM has received consulting fees from SAGE Therapeutics. None of the other authors has any conflict of interest to disclose. We confirm that we have read the Journal’s position on issues involved in ethical publication and affirm that this report is consistent with those guidelines.

## Authors’ contributions

DP collected the data, conceived of the study, participated in its design and performed the statistical analysis, and wrote the manuscript. BF, GM, BMC, SA, and SAM helped with conceptualization of the study and drafting of the manuscript. AF, HL, and CL helped with the data collection and conceptualization of the study, and critically revising the manuscript. MJS helped with study conceptualization, analysis of the data, and drafting of the manuscript. JC helped with data collection, design and coordinated the study and, helped to draft the manuscript. All authors read and approved the final manuscript.
